# Fitness to practise sanctions in UK doctors are predicted by poor performance at MRCGP and MRCP(UK) assessments: data linkage study

**DOI:** 10.1186/s12916-018-1214-4

**Published:** 2018-12-07

**Authors:** Richard Wakeford, Kasia Ludka, Katherine Woolf, I. C. McManus

**Affiliations:** 1 Hughes Hall, University of Cambridge, Cambridge, CB1 2EW UK; 2 Onet, ul., Marszałkowska 76, Warsaw, Poland; 3 Research Department of Medical Education, UCL Medical School, University College London, Gower Street, London, WC1E 6BT UK

**Keywords:** Fitness to practise, MRCGP, MRCP(UK), Knowledge assessments, Clinical assessments, Postgraduate examinations, GMC sanctions

## Abstract

**Background:**

The predictive validity of postgraduate examinations, such as MRCGP and MRCP(UK) in the UK, is hard to assess, particularly for clinically relevant outcomes. The sanctions imposed on doctors by the UK’s General Medical Council (GMC), including erasure from the Medical Register, are indicators of serious problems with fitness to practise (FtP) that threaten patient safety or wellbeing. This data linkage study combined data on GMC sanctions with data on postgraduate examination performance.

**Methods:**

Examination results were obtained for UK registered doctors taking the MRCGP Applied Knowledge Test (AKT; *n* = 27,561) or Clinical Skills Assessment (CSA; *n* = 17,365) at first attempt between 2010 and 2016 or taking MRCP(UK) Part 1 (MCQ; *n* = 37,358), Part 2 (MCQ; *n* = 28,285) or Practical Assessment of Clinical Examination Skills (PACES; *n* = 27,040) at first attempt between 2001 and 2016. Exam data were linked with GMC actions on a doctor’s registration from September 2008 to January 2017, sanctions including Erasure, Suspension, Conditions on Practice, Undertakings or Warnings (ESCUW). Examination results were only considered at first attempts. Multiple logistic regression assessed the odds ratio for ESCUW in relation to examination results. Multiple imputation was used for structurally missing values.

**Results:**

Doctors sanctioned by the GMC performed substantially less well on MRCGP and MRCP(UK), with a mean Cohen’s *d* across the five exams of − 0.68. Doctors on the 2.5th percentile of exam performance were about 12 times more likely to have FtP problems than those on the 97.5th percentile. Knowledge assessments and clinical assessments were independent predictors of future sanctions, with clinical assessments predicting ESCUW significantly better. The log odds of an FtP sanction were linearly related to examination marks over the entire range of performance, additional performance increments lowering the risk of FtP sanctions at all performance levels.

**Conclusions:**

MRCGP and MRCP(UK) performance are valid predictors of professionally important outcomes that transcend simple knowledge or skills and the GMC puts under the headings of conduct and trust. Postgraduate examinations may predict FtP sanctions because the psychological processes involved in successfully studying, understanding and practising medicine at a high level share similar mechanisms to those underlying conduct and trust.

## Background

Perhaps the most serious event in a UK doctor’s professional life is to be investigated by the General Medical Council (GMC) because of concerns about their fitness to practise (FtP). FtP concerns are investigated in a quasi-legal fashion; the proceedings can be very stressful [1] and can result in sanctions, of which the most serious are to be ‘struck off’ (erased) or suspended from the medical register (LRMP; List of Registered Medical Practitioners). About 1% of doctors on the medical register have been sanctioned by the GMC for FtP issues at some point in their career [2]. There is considerable interest among the profession and patients in understanding why doctors are sanctioned and identifying these doctors early before personal, professional and patient harms occur. More generally, there are also current debates about the purpose and validity of large-scale medical examinations. Postgraduate examinations in the UK traditionally have clinical assessments, although these are expensive to run and there are concerns about reliability, whereas in the US, no postgraduate examination has a clinical component, being restricted to written, knowledge assessments. At the undergraduate level, all UK medical schools have clinical assessments as a part of finals, and that will continue to be the case with the development by the GMC of the UK Medical Licensing Assessment (UKMLA)[3]. The format of licencing examinations, though, continues to be controversial [4]. In the US, the National Board of Medical Examiners (NBME), in 2004, introduced the Step 2 Clinical Skills examination into the United States Medical Licensing Examination (USMLE). That examination is controversial, a high-profile paper, supported by a petition with 16,000 signatories, argued for its abolition, for multiple reasons, including excessive cost and the absence of evidence of improvements in patient safety or public trust in physicians [5].

The GMC has a statutory duty to quality assure the UK medical workforce by two mechanisms. *Revalidation* requires all doctors to demonstrate on a regular basis that they are up to date and fit to practise in their chosen field and able to provide a good level of care [6]. The *fitness to practise (FtP) procedures* are invoked following a complaint about a doctor that raises a concern about their fitness to practise. Complaints are firstly investigated by the GMC, with the investigation results being reviewed by two GMC Case Examiners, who refer cases deemed sufficiently serious to the Medical Practitioners Tribunal Service (MPTS), sometimes by way of the GMC’s Investigation Committee. Although funded by the GMC, the MPTS acts independently of it and reports to Parliament. The MPTS will decide whether or not to impose a sanction, which include, in decreasing order of severity, the doctor being *erased* or *suspended* from the medical register, having *conditions* imposed on their registration, a doctor agreeing to *undertakings* or a doctor being given a *warning*. We refer to these sanctions collectively as ESCUW (Erasure, Suspension, Conditions, Undertakings or Warnings) [7]. The GMC FtP procedures are governed by the Medical Act 1983 and the GMC (Fitness to Practise) Rules 2004, under which a doctor’s fitness to practise can be impaired due to misconduct, deficient performance, a criminal conviction or caution, adverse physical or mental health, determination by regulatory bodies in the British Isles or overseas or not having the necessary knowledge of English [6].

The fact that the FtP procedures are entirely independent of Royal College postgraduate examinations provides an opportunity to assess whether a doctor’s knowledge, skills and professional behaviour assessed under examination conditions relate to an entirely separate assessment of a doctor’s performance in the entire context of their practice and professional behaviour. In this study, we use a data linkage study to show that doctors found to be impaired under FtP procedures also perform far less well in the various knowledge-based and clinical examinations of the MRCGP (Membership of the Royal College of General Practitioners) and the MRCP(UK) (Membership of the Royal Colleges of Physicians of the United Kingdom). A preliminary version of these analyses using more restricted data was presented by Ludka [8] as a part of her PhD thesis [9].

Postgraduate examinations in the UK are central to ensuring the quality of trainees who become specialists in hospital care or general practice, assessing high-level knowledge, clinical skills and professional behaviours. A surprisingly common informal critique of such examinations is that they ‘only assess knowledge’ and ‘only test the ability to pass examinations’, and a recent Royal College of Anaesthetists report said that ‘Professional examinations were … felt to not always be relevant to contemporary clinical practice’ [10]. The implication is that postgraduate examinations, and medical examinations more generally, are somehow merely some form of academic game that bear little relationship to the real world of clinical practice.

If medical examinations are indeed worthwhile then, as with all medical assessments, undergraduate and postgraduate, they need to have demonstrable validity [11–15], although definitions of validity are evolving. A ‘holy grail’ for postgraduate assessments is to relate examination performance to important outcomes in terms of patient morbidity or to the censure (sanctioning) of doctors for unprofessional behaviour. The only study of exam performance in relation to patient outcomes is the 2014 study by Norcini et al. [16] in the USA, showing that poorer performance on the US Medical Licensing Examination (USMLE) by international medical graduates (IMGs) was associated a decade later with a higher mortality in patients treated by those doctors. Although the authors do not directly discuss the issue of causality, they implicitly suggest a causal relationship by saying that the study provides evidence for the validity of the exam, they emphasise the long time interval between the exam and the clinical outcomes, they comment on the results being ‘consistent with the growing literature suggestion that national high-stakes examinations have a positive relationship with patient outcomes’, they say the findings ‘support the use of the examination as an effective screening strategy for licensure’ and they comment that gathering validity evidence is ‘challenging … because it is not possible to randomise to treatment …’ [16]. Several other studies have looked at the link between exam performance and FtP/censure outcomes for doctors. Poor performance in the certification examination of the ABIM (American Board of Internal Medicine), a knowledge examination, was shown in 2008 to relate to higher risks of unprofessional behaviour [17]. A 2017 study of USMLE in US medical graduates found that lower performance on Step 1 (biomedical sciences) and Step 2 CK (clinical knowledge) exams was associated with a higher likelihood of state medical board sanctions [18]. These ABIM and USMLE studies considered only knowledge assessments rather than clinical assessments. However, a study of USMLE Step 2 Data Gathering and Data Interpretation scores predicted supervisor ratings of history taking and physical examination during residency [19]. In the UK, Tiffin et al. in 2017 found that lower scores on the PLAB examination (Professional and Linguistics Assessments Board examination, the GMC’s licencing examination for IMGs wishing to work in the UK), particularly the clinical assessment (Part 2), predicted the likelihood of sanctioning by the GMC [20].

Previous studies of FtP have therefore concentrated on licencing assessments and mostly but not entirely have considered knowledge assessments. In this study, we assess the association of poor performance on high-level UK postgraduate exams with the likelihood of FtP sanctions, and in particular, we consider the separate roles of both clinical and knowledge assessments. The study therefore differs from previous work in emphasising national postgraduate examinations, in comparing the value of knowledge and clinical assessments in predicting poor performance and in considering both UK graduates and non-UK graduates.

## Method

Analyses were carried out separately for MRCGP and MRCP(UK), with examination performance being linked to the FtP sanctions recorded on the publically available version of the LRMP. A small number of candidates take both MRCGP and MRCP(UK) [21], but analyses for present purposes were conducted separately. For both analyses, examination performance was based on marks at the first attempt, which are the most useful predictors of subsequent performance [22]. Marks at first attempt are approximately normally distributed, whereas marks at second and later attempts are difficult to interpret as a result of failure at first or other previous attempts. Pass marks vary between diets (sittings) of an exam due to variation in question difficulty, and therefore, all marks were firstly expressed as marks above or below the pass mark. They were then converted to *z*-scores (mean 0; SD 1), which allows a direct comparison of different examinations which have different marking schemes. For MRCP(UK) Parts 1 and 2, diets from 2009/1 to 2010/1 onwards statistical equating were used for standard setting, with marks expressed relative to a fixed pass mark [23]. For earlier diets and other exams, the use of *z*-scores does not provide a full and complete equating but in practical terms is a pragmatic approach.

### MRCGP

The MRCGP examination is in two parts, typically taken in the second and third year of specialty training, which are in the fourth and fifth year after qualification. The AKT (Applied Knowledge Test) is assessed by a 190-min, mainly multiple-choice, assessment with 200 questions, largely in the one-from-five format, and the CSA (Clinical Skills Assessment) is an assessment of clinical skills, assessed by means of a 3-h examination involving cases played by simulated patients (actors) across 13 stations in a simulated surgery (clinic). Details can be seen on the RCGP website [24]. For the AKT, the primary dataset was for 35,368 candidates who took the exam between 2007 and 2016, of whom 27,561 were at their first attempt. For the CSA, the primary dataset was for 23,158 candidates taking the examination between 2010 and 2016, of whom 17,365 were on their first attempt.

### MRCP(UK)

The MRCP(UK) examination is in three parts, Part 1, Part 2 and PACES (Practical Assessment of Clinical and Examination Skills), which typically are taken in the second to fourth years after qualifying. Parts 1 and 2 during the study period were multiple-choice-based knowledge assessments [23], lasting 6 and 9 h, with 200 and 270 best-of-five questions. PACES and, its successor, nPACES introduced in 2009 [25] are clinical examinations assessing physical examination, diagnosis, management, history-taking and communication. The primary dataset contained results for 44,314 candidates of whom 37,358 were taking Part 1 for the first time in the diets from 2002/2 (i.e. the second diet [sitting] of 2002) to 2016/3, for 28,285 candidates taking Part 2 for the first in the diets from 2002/3 to 2016/3 and 27,040 taking PACES for the first time. nPACES has a pass mark which must be achieved on each of seven separate skills, and so for ease of analysis here, a single composite score was calculated which was equated to that in PACES. Equating was initially carried out linearly using data acquired at the piloting stage of nPACES when examiners assessed candidates using the marking schemes for both PACES and nPACES and was subsequently validated by showing that pass rates did not differ for the old and new marking schemes.

### LRMP

The complete LRMP is provided on subscription and was downloaded at monthly intervals from Sept 2008 to Jan 2017 and any practitioners with sanctions noted. Linkage to MRCGP and MRCP(UK) was by means of the GMC number, the unique reference number for all doctors registered in the UK. Doctors were recorded as having any of the five ESCUW FtP sanctions at any point in the dataset, and the overall binary ESCUW variable recorded whether or not doctors had any sanctions at any time. Detailed reasons for sanctions are not available on the LRMP, nor does it contain information about whether a doctor is currently under investigation, unless they have been suspended temporarily while the investigation takes place. Complaints and FtP issues are known to be more frequent amongst male doctors, BME (Black and minority ethnic) doctors and doctors who graduated from a non-UK medical school [26].

### Demographics of doctors

The sex of doctors, along with whether they were graduates of UK or non-UK medical schools, was obtained from the LRMP. Ethnicity of doctors is not included in LRMP, but self-reported ethnicity is available for a majority of doctors in the MRCGP and MRCP(UK) databases and for present purposes was coded as White vs BME. Variation in FtP sanctions by sex, ethnicity and place of qualification is not immediately relevant to assessing the extent to which examination results predict FtP issues, since written examinations are marked independently of knowledge of sex, ethnicity or place of qualification (although all three show a relationship to examination performance [21]). However, our analyses of the relationship of FtP sanctions and performance on written and clinical examinations separately within groups based on sex, ethnicity and place of qualification will show that confounding cannot explain the association that we find. Machine-marked knowledge assessments can show differential item functioning (DIF), whereby item performance relates to sex or ethnicity, and analyses of MRCP(UK) Part 1 suggest that differential item performance in UK graduates in relation to sex or ethnicity is extremely rare [27]. In contrast, DIF does occur when UK and non-UK graduates are compared [23], but probably relates to differential training and clinical experience.

### Statistical methods

Statistical analysis used *IBM SPSS v24.0* and *R v3.4.4*. Graphical analyses used *IBM SPSS* and the *ggplot2* package in *R* [28].

Simple comparisons of the mean marks of doctors with or without ESCUW used *t* tests, with effect sizes calculated as Cohen’s *d* and AUC, the area under the ROC (receiver operating characteristic) curve. ROC analyses are carried out to assess how effective the examination results would be, were they to be used as a diagnostic test for subsequent ESCUW, i.e. to see the relationship between *specificity* (the true positive rate) and *1-sensitivity* (the false positive rate) as a result of using different thresholds. For ROCs, the area under the curve (AUC) is a measure of the effectiveness of a diagnostic test, greater areas indicating better ability to predict outcomes. Comparison of ROC curves and the calculation of AUCs and their standard errors, as well as comparison of AUCs between assessments, were carried out using the *pROC()* function in R [29].

Logistic regressions were used to model the binary outcome of ESCUW in relation to predictor variables expressed as standardised (*z*) scores with a mean of 0 and standard deviation (SD) of 1, so that *b* values give the increased log_e_(OR) [OR = odds ratio] for a 1 SD change in an examination score. ORs for a particular exam were expressed as the increased odds of ESCUW for a doctor on the 2.5th percentile of exam marks in relation to a doctor on the 97.5th percentile, other examinations being at mean performance.

Multiple logistic regression assessed the log_e_(OR) of ESCUW in relation to MRCGP AKT and CSA, and to MRCP(UK) Parts 1, 2 and PACES, to assess the relative prediction from different examination types. Missing values were present for structural reasons, doctors who failed one part of MRCP(UK) typically not taking later parts, but results in both exams are also missing because of truncation within the time window used, some exams being taken outside of the time window, i.e. before data collection began or in the future. Missing exam results for the multiple regression were imputed 100 times using the Multiple Imputation package in *IBM SPSS* under the assumption that data are missing at random (MAR).

### A note on the interpretation of log odds ratios

Raw data can be expressed in terms of the *probability*, *p*, which is the proportion of cases having a sanction, and hence, the probability of not having a sanction is (1 − *p*)*.* The *odds* of having a sanction is *p*/(1 − *p*). Logistic regression models *logit(p)* where *logit*(*p*) = *log*_*e*_(*odds*) = *log*_*e*_(*p*/(1 − *p*)), *log*_*e*_() indicates natural logarithms to base *e* (with *e* being Euler’s number, 2.71828…). Logistic regression is used because probabilities are bounded by the values 0 and 1, and simple regression models with probabilities as a dependent variable can predict nonsensical probabilities of greater than 1 or less than 0. In contrast *logit(p)* has a range from minus infinity to plus infinity. For similar reasons, it mostly makes little theoretical sense to plot *p* against a predictor as the relationship is necessarily curvilinear, *p* being constrained to the range 0 to 1. Likewise, it rarely makes sense to plot odds against a predictor variable, particularly when *p* values are low (the so-called rare disease case) since as *p* approaches 0 and odds are calculated as *p*/(1 − *p*), so 1 − *p* approaches 1 and therefore odds ≈ *p*/1 = *p*, also making curvilinearity inevitable and difficult to interpret. Interpreting *log*_*e*_(odds) is not always intuitive but is simpler when *p* values are small. Logistic regression equations say that *logit*(*p*) *= a + b.x*, where *a* is a constant and *b* a multiplier of a predictor variable, *x*. For small *p*, the equation becomes *log(p) = a + b.x*, and *b* indicates the *multiplicative* change in *p* for a one-step change in *b.* As a simple example, consider probabilities of 0.1, 0.01, 0.001 and 0.0001, predictors of values 1, 2, 3 and 4, and let logs be to base 10, so that log_10_(p) is − 1, − 2, − 3 and − 4. A one-step increase in *x*, e.g. from 1 to 2, results in a decrease in log(p) of − 1, and hence, *p* is reduced by a factor of 10. Because *a + b.x* is a linear model, then were an intervention to reduce *x* by 1 then *all* probabilities would be reduced by a factor of 10, whether they started at 0.1 or 0.0001, indicating a common mechanism or process.

## Results

### ESCUW

Considering all the 380,583 doctors in the LRMP records at 1 Jan 2017, there were 6158 doctors who had ESCUW during the study period (1.62%), 680 (0.23%) erased, 2250 (0.59%) suspended, 2871 (0.75%) with conditions, 1263 (0.33%) with undertakings and 1735 (0.46%) with warnings. Three thousand eight hundred seventy-eight (63.0%) of the 6158 doctors with ESCUW had only one FtP sanction, the remainder having two (1776; 28.8%), three (468; 7.6%), four (35; 0.6%) or five (1; 0.02%) sanctions. The log_e_(OR) of a doctor having ESCUW was analysed using a multiple logistic regression on the entire LRMP in terms of a doctor’s sex, place of qualification (UK vs others), years since qualification and years since qualification not spent on the GMC Register (presumably for IMGs due to time spent working outside the UK before arrival in the UK). Doctors who had been on the LRMP for longer were more likely to have ESCUW, rising from 0.5% of doctors in the first decade after graduation to 1.3% in the second decade, 2.0% in the third decade, 2.8% in the fourth decade and 3.1% in the fifth decade, presumably due to increasing opportunity for problems to arise. The majority of doctors taking MRCGP or MRCP(UK) during the time period of this study were in the first decade or two after qualifying (median of 11 years since qualification).

Multiple logistic regression showed that those with ESCUW were 2.73× more likely to be male (log_e_(OR) = 1.004, SE = 0.034, OR = 2.730×), to have been qualified longer (log_e_(OR) = 0.065 per decade, SE = .008, OR = 1.067× per decade), not to have qualified in the UK (log_e_(OR) = 0.304, SE = 0.033, OR = 1.355×) and to have spent more time not on the GMC Register (log_e_(OR) = 0.206 per decade, SE = 0.023, OR = 1.228× per decade). Note that the vast majority of doctors who had spent longer not on the GMC Register were those who had qualified outside of the UK. It should also be emphasised that ethnicity is not available in the LRMP.

### MRCGP

MRCGP results were available for 27,561 doctors who had taken AKT at the first attempt during the study period, with the 423 (1.53%) having ESCUW scoring significantly lower on the exam than those without ESCUW, the Cohen’s *d* effect size being − 0.734 (Table 1). Similarly, of 17,365 doctors who had taken CSA at the first attempt, 238 (1.38%) had ESCUW and had scored significantly lower on the exam, with a Cohen’s *d* of − 0.805 (Table 1). Simple logistic regressions showed significant effects for both AKT and CSA (Table 2).

**Table 1:** Descriptive statistics for examination performance of candidates with and without ESCUW; means and standard deviations, *t* test results and Cohen’s *d* for effect size. All differences are significant with *p* < 0.001

Exam	No ESCUW	ESCUW	Sig	Cohen’s *d*
MRCGP: AKT	0.011 (0.993) *N* = 27,138	− 0.720 (1.165) *n* = 423	*t*(27,559) = 14.99	− 0.734
MRCGP: CSA	0.011 (0.995) *N* = 17,127	− 0.790 (1.073) *N* = 238	*t*(17,363) = 12.33	− 0.805
MRCP(UK): Part 1	0.007 (0.997) *N* = 36,934	− 0.610 (1.051) *N* = 424	*t*(37,356) = 12.64	− 0.617
MRCP(UK): Part 2	0.005 (0.999) *N* = 28,011	− 0.528 (0.956) *N* = 274	*t*(28,283) = 8.80	− 0.536
MRCP(UK): PACES	0.007 (0.996) *N* = 26,751	− 0.686 (1.126) *N* = 289	*t*(27,038) = 11.76	− 0.696

**Table 2:** Simple logistic regressions of ESCUW (vs no ESCUW) on performance in the individual components of each examination. The table shows *b* = log_e_(OR), OR (i.e. *e*^*b*^) and its 95% confidence interval, and the odds ratio for comparing the likelihood of ESCUW in candidates at the 2.5th percentile (i.e. 2 SDs below the population mean examination performance) and at the 97.5th percentile (i.e. 2 SDs above the mean performance). All *b* values for predictors are significant with *p* < 0.001

Exam	Constant	*b* = log_e_(OR)	OR = e^b^ (95% CI)	OR for ESCUW 2.5th percentile to 97.5th percentile
MRCGP: AKT *n* = 27,561	− 4.364	− 0.611	0.543× (0.500, 0.589)	11.52×
MRCGP: CSA *n* = 17,365	− 4.533	− 0.692	0.501× (0.447, 0.561)	15.93×
MRCP(UK): Part 1 *n* = 37,358	− 4.645	− 0.597	0.550× (0.501, 0.605)	10.89×
MRCP(UK): Part 2 *n* = 28,285	− 4.786	− 0.589	0.555× (0.487, 0.633)	10.55×
MRCP(UK): PACES *n* = 27,040	− 4.716	− 0.586	0.557× (0.504, 0.615)	10.42×

Multiple logistic regression of ESCUW on both AKT and CSA used 100 multiple imputations for the 27,651 candidates. EM (expectation maximisation) estimation of means and SDs showed no difference in overall performance of imputed and non-imputed cases, missing CSA results being due to candidates not yet having taken the exam. The multiple logistic regression showed that AKT and CSA both had independent predictive effects after taking the other into account, with the effect of CSA being greater than that for AKT and the 95% confidence intervals for the ORs not overlapping (Table 3: AKT OR = 0.736: 95% CI 0.659 to 0.821; CSA OR = 0.566: 95% CI = 0.495 to 0.647). For completeness, the equivalent ORs on the raw, non-imputed dataset were calculated (AKT OR = 0.774: 95% CI 0.678 to 0.885; CSA OR = 0.576: 95% CI = 0.502 to 0.660) and are very similar to the imputed results.

**Table 3:** Multiple logistic regression of ESCUW (vs no ESCUW) on performance on the separate examination components of each examination. Data are necessarily structurally missing as candidates who do not pass one component do not continue to take later components, so that missing values have been replaced using 100 multiple imputations, and there is also right and left truncation. The table shows the effect for each exam component, after partialling out the effects of all other components. The columns show *b =* log_e_(OR), OR (i.e. e^b^) and the OR for comparing the likelihood of ESCUW in candidates at the 2.5th percentile (i.e. 2 SDs below the population mean examination performance) and at the 97.5th percentile (i.e. 2 SDs above the population mean), performance of all other assessments being taken as at the mean. All *b* values for predictors are significant with *p* < 0.001

Exam	Component	*b* = log_e_(OR)	OR = e^b^ (95% CI)	OR for ESCUW 2.5th percentile vs 95th percentile
MRCGP *n* = 27,561	Constant	− 4.482	0.011×	
	AKT	− 0.307	0.736× (0.659, 0.821)	3.33×
	CSA	− 0.569	0.566× (0.495, 0.647)	9.30×
MRCP(UK) *n* = 44,314	Constant	− 4.726	0.009×	
	Part 1	− 0.206	0.814× (0.703, 0.943)	2.24×
	Part 2	− 0.309	0.734× (0.617, 0.874)	3.36×
	PACES	− 0.496	0.609× (0.538, 0.689)	6.99×

**Fig. 1:**
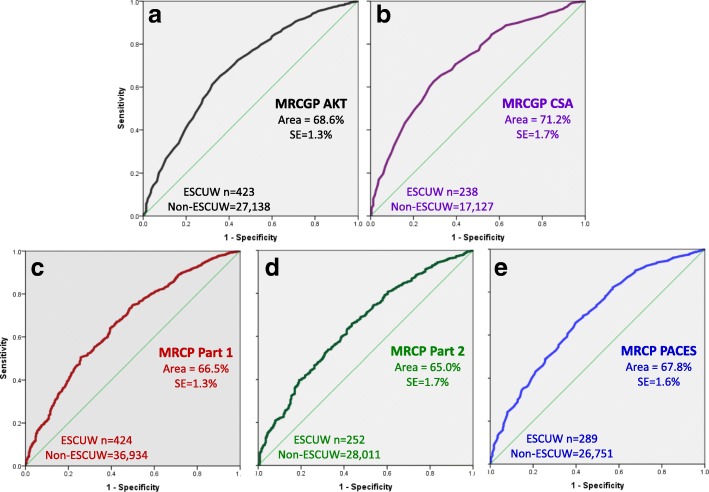
ROC curves for predicting ESCUW on the basis of examination results for the two MRCGP and three MRCP(UK) assessments. The vertical axis shows the sensitivity, and the horizontal axis (1-Specificity). The area under the curve ('Area') and its standard error ('SE'), along with the number of cases of ESCUW and non-ESCUW are shown within each plot

Areas under the ROC curve were 68.6% (SE 1.3%) and 71.2% (SE 1.7%) for the AKT and CSA assessments, respectively (see Fig. 1a, b). A paired analysis of AUCs under the AKT and CSA curves using the *roc.test()* function in *pROC* showed a significant difference (*z* = 2.82, *z* = 0.0048), with AUC estimates in the paired data of 0.666 and 0.713 for AKT and CSA, respectively, showing that the CSA better predicts ESCUW than AKT.

### MRCP(UK)

After merging, the database had 44,314 doctors who had taken Part 1 (*n* = 37,358), Part 2 (*n* = 28,285) or PACES (*n* = 27,040) at a first attempt and were on the LRMP and so had data on ESCUW. Twenty thousand two hundred ninety-nine doctors had taken all three MRCP(UK) parts, 7771 had taken two parts and 20,299 had taken only one part.

Of 37,358 doctors who were on their first attempt at Part 1, 423 (1.13%) had ESCUW and had significantly lower scores on the exam, Cohen’s *d* being − 0.617 (Table 1). In a simple logistic regression, standardised Part 1 scores significantly predicted ESCUW (log_e_(OR) = − 0.597, SE = 0.048, OR = 0.550× per SD). Multiple logistic regression taking into account sex, ethnicity, UK qualification, decades since qualification and decades not on the UK Register showed that Part 1 scores were still significant predictors of ESCUW (log_e_(OR) = − 0.376, SE = 0.052, OR = 0.687× per SD).

For first attempt at Part 2, there were 28,285 doctors of whom 274 had ESCUW (0.97%), and they had significantly lower marks with an effect size of − 0.536. On its own, Part 2 score predicted ESCUW (log_e_(OR) = − 0.589 per SD, SE = 0.066, OR = 0.555× per SD). After taking sex, UK qualification, decades since qualification and decades not on the UK Register into account, Part 2 scores were still significant predictors of ESCUW (log_e_(OR) = − 0.379 per SD, SE = 0.074, OR = 0.685× per SD).

Of 27,040 doctors taking PACES for the first time, 289 (1.07%) had ESCUW and had scored significantly lower on the exam with Cohen’s *d* = − 0.696 (Table 1). PACES score alone predicted ESCUW (log_e_(OR) = − 0.588 per SD, SE = 0.051, OR = 0.555× per SD), and the effect remained highly significant after taking into account sex, UK qualification, decades since qualification and decades not on the UK Register (log_e_(OR) = − 0.356 per SD, SE = 0.063, OR = 0.700× per SD).

Data for Part 2 and PACES were missing where candidates had failed earlier exams (Part 1 or Part 2) or had taken exams before the time window in which data were available or had not yet had time to take later exams. The former particularly needs taking into account for an analysis of the relative predictive importance of the separate parts of the MRCP(UK) exam. The EM algorithm was used to estimate means and SDs of all the 44,314 candidates, irrespective of which parts of the exam they had taken. The EM estimated mean (SD) on *z*-score-transformed marks for Part 1 was − 0.1045 (.993), for Part 2 was − 0.168 (1.040) and for PACES was − 0.085 (1.016), compared with means of 0 (SD 1) in the raw *z*-score-transformed data. Missing data therefore were biased, not least as candidates who fail a part at a first attempt are less likely to take further parts, but if they had, they would have performed less well than those passing parts at a first attempt [8].

Multiple logistic regression was carried out on 100 sets of data with missing values imputed. Table 3 shows that all three examinations had significant, independent predictions of ESCUW (Part 1: OR = 0.814, 95% CI = 0.703 to 0.943; Part 2: OR = 0.734, 95% CI = 0.617 to 0.874; PACES: OR = 0.609, 95% CI = 0.538 to 0.689). PACES had a significantly larger prediction of ESCUW (log_e_(OR) = − 0.496 per SD; 95% CI − 0.619 to − 0.373) than did Part 1 (log_e_(OR) = − 0.206 per SD; 95% CI = − 0.353 to − 0.059), the confidence intervals not overlapping. The independent predictive effect of Part 2 was between that of PACES and Part 1. Analysis of the non-imputed raw dataset based on 20,299 complete cases found broadly similar effects to that of the imputed data set (Part 1: OR = 1.007; 95% CI = 0.820 to 1.237; Part 2: OR = 0.745; 95% CI = 0.607 to 0.916; PACES: OR = 0.655; 95% CI = 0.568 to 0.756), with significant effects for Part 2 and PACES (*p* = 0.005 and *p* < 0.001) but not for Part 1 (*p* = 0.945). The non-significant result for Part 1 presumably occurs because it is heavily range restricted, only candidates passing all three parts being included, and Part 2 correlating with Part 1 and accounting for Part 1’s variance.

The areas under the ROC curves were 66.5% (SE 1.3%), 65.0% (SE 1.7%) and 67.8% (SE 1.6%) for Parts 1, 2 and PACES, respectively (see Fig. 1c–e). A paired analysis of AUCs for Part 1, Part 2 and PACES curves using the *roc.test()* function in *pROC* showed a significant difference between Part 1 and PACES (*z* = 2.39, *p* = 0.017, AUCs for paired data = 0.593 and 0.653) but not between Part 1 and Part 2(*z* = 1.49, *p* = 0.14, AUCs for paired data = 0.645 and 0.676) or Part 2 and PACES (*z* = 1.88, *p* = 0.060, AUCs for paired data = 0.602 and 0.635). PACES is therefore a better predictor of ESCUW than either Part 1 or Part 2.

Unpaired comparison of ROCs for the MRCGP and MRCP(UK) assessments showed no significant differences in AUCs between the knowledge tests (AKT vs Part 1, *p* = 0.27) or the clinical assessments (CSA vs PACES, *p* = 0.14).

### Overall estimates of effect size

It is convenient to have a simple overall estimate of the relationship of exam results to the likelihood of FtP issues. For Table 1, the unweighted mean of the five effect sizes is − 0.678 (range = − 0.536 to − 0.805), and for Table 2, the unweighted mean of the five odds ratios comparing candidates at the 2.5th and 97.5th performance percentiles is 11.86×.

### The detailed relationship of FtP sanctions to examination performance

**Fig. 2:**
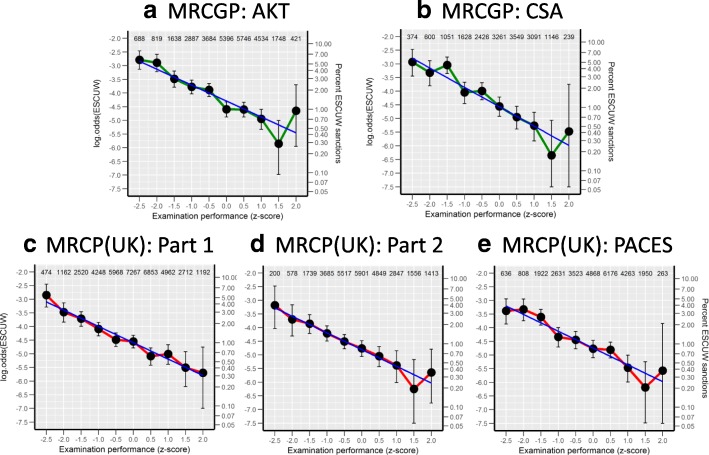
The abscissa shows the *z*-score of performance at the first attempt of candidate performance for each examination, divided into ten equal groups from − 2.5 to + 2 SDs, with *N* for each group shown at the top. The left-hand ordinate shows log_e_(odds) for an FtP sanction (ESCUW), and the right-hand ordinate shows the percentages of candidates with ESCUW sanctions. Note that although scales correspond exactly, the left-hand ordinate is linear and the right-hand ordinate is non-linear. The fitted line (blue) is linear on the log(odds) scale. Error bars show 95% confidence intervals, and almost all 95% confidence intervals include the fitted line

FtP sanctions are clearly related to lower examination performance. However, the shape of that relationship has theoretical implications. Figure 2 shows the log_e_(OR) of a doctor having FtP sanctions in relation to the standardised examination performance. All five assessments show a very similar pattern, a key feature of which is the linearity of the relationship between log_e_(OR) and examination performance, within the limits of random variation, that is visible within both high-scoring and low-scoring candidates. Increments in performance at all levels of performance therefore result in decrements in FtP sanctions. In view of the theoretical importance of the linearity for each of the five predictors, we have included quadratic and cubic components of examination performance into the model, and in no case were the additional improvements in fit significant at the *p* < 0.05 level. The linearity of the relationships between log_e_(OR) and examination performance in Fig. 2 are therefore a robust and an intriguing finding.

### ESCUW in subgroups based on place of graduation, sex and ethnicity

Place of Primary Medical Qualification (PMQ; coded as UK or elsewhere), sex and ethnicity are known to be related to FtP sanctions, and PMQ, sex and ethnicity also relate to performance of postgraduate medical examinations. It is therefore important to ensure that the predictive effects of postgraduate performance are not confounded by demographic differences. Table 4 shows, for both MRCGP and MRCP(UK), the performance of ESCUW and non-ESCUW doctors broken down by UK/non-UK graduate, male/female and white/BME (with ethnicity not broken down in non-UK graduates as *N*s of white doctors were small). In all sub-groups on all examinations, the ESCUW doctors perform less well, although differences are not always individually significant due to small sample sizes. Analyses of variance of the five examinations showed that UK graduates and White doctors in each case performed better (*p* < 0.001 in each case). Sex differences were more variable with men performing better on MRCP(UK) Parts 1 and 2 but women performing better on MRCGP AKT and CSA and MRCP(UK) PACES. Despite all such differences, the ESCUW doctors performed less well overall for all examinations (*p* < 0.001), with no interactions with UK graduation, sex or ethnicity. The mean overall Cohen’s *d* was − 0.49, which is a little less than the values in Table 1 because variance between groups has been removed.

**Table 4:** Mean performance of candidates (SD) divided by UK/non-UK graduate; male/female and White/non-White. Non-UK graduates are not divided by ethnicity as there are very small numbers of White graduates

Mean (SD)/*N*	MRCGP		MRCP(UK)		
	AKT	CSA	Part 1	Part 2	PACES
UK graduates, male, White					
No ESCUW	0.297 (0.815) *N* = 4306	0.251 (0.750) *N* = 2755	0.427 (0.891) *N* = 6403	0.459 (0.987) *N* = 5160	0.369 (0.776) *N* = 4782
ESCUW	− 0.067 (1.071) *N* = 50	0.003 (0.627) *N* = 33	0.137 (0.894) *N* = 51	0.212 (1.213) *N* = 40	0.210 (0.893) *N* = 41
Sig	*t*(4,354) = 3.13 *p* = 0.002	*t*(2,786) = 1.89 *p* = 0.058	*t*(6,452) = 2.31 *p* = 0.021	*t*(5,198) = 0.157 *p* = 0.117	*t*(4,821) = 0.192 *p* = 0.192
Cohen’s *d*	− 0.45	− 0.33	− 0.33	− 0.20	− 0.20
UK graduates, female, White					
No ESCUW	0.447 (0.781) *N* = 9381	0.591 (0.695) *N* = 5950	0.220 (0.839) *N* = 9301	0.246 (0.886) *N* = 6950	0.491 (0.728) *N* = 6429
ESCUW	− 0.264 (1.173) *N* = 46	0.139 (0.981) *N* = 28	− 0.165 (0.685) *N* = 30	− 0.170 (0.823) *N* = 19	0.235 (0.669) *N* = 19
Sig	*t*(9,425) = 6.13 *p* < 0.001	*t*(5,976) = 3.43 *p* = 0.001	*t*(9,329) = 2.56 *p* = 0.012	*t*(6,967) = 2.05 *p* = 0.041	*t*(6,446) = 1.53 *p* = .126
Cohen’s *d*	− 0.91	− 0.65	− 0.46	− 0.40	− 0.33
UK graduates, male, Non-White					
No ESCUW	− 0.161 (0.954) *N* = 2699	− 0.206 (0.839) *N* = 1762	0.211 (0.968) *N* = 4576	0.125 (0.995) *N* = 3607	0.072 (0.877) *N* = 3628
ESCUW	− 0.576 (1.061) *N* = 95	− 0.778 (0.938) *N* = 59	− 0.334 (1.044) *N* = 88	− 0.280 (0.939) *N* = 55	− 0.394 (1.076) *N* = 53
Sig	*t*(2,792) = 4.15 *p* < .001	*t*(1,819) = 5.13 *p* < .001	*t*(4,662) = 5.24 *p* < 0.001	*t*(3,660) = 3.00 *p* = 0.003	*t*(3,679) = 3.84 *p* < 0.001
Cohen’s *d*	− 0.44	− 0.68	− 0.56	− 0.52	− 0.53
UK graduates, female, Non-White					
No ESCUW	− 0.035 (0.907) *N* = 3857	.141 (.779) *N* = 2492	.070 (.901) *N* = 5391	−.051 (.889) *N* = 3903	.239 (.812) *N* = 3806
ESCUW	− 0.606 (0.934) *N* = 24	− 0.361 (1.006) *N* = 16	− 0.399 (0.824) *N* = 27	− 0.574 (0.422) *N* = 19	0.055 (0.752) *N* = 20
Sig	*t*(3,879) = 3.07 *p* = 0.002	*t*(2,506) = 2.57 *p* = 0.010	*t*(5,416) = 2.70 *p* = 0.002	*t*(3,920) = 2.56 *p* = 0.010	*t*(3,824) = 1.01 *p* = 0.312
Cohen’s *d*	− 0.63	− 0.64	− 0.53	− 0.54	− 0.23
Non-UK graduates, male					
No ESCUW	− 0.774 (1.021) *N* = 3281	− 1.238 (0.879) *N* = 1966	− 0.482 (1.045) *N* = 6763	− 0.487 (0.941) *N* = 5462	− 0.818 (1.008) *N* = 5483
ESCUW	− 1.085 (1.182) *N* = 162	− 1.400 (0.925) *N* = 76	− 0.889 (1.047) *N* = 182	− 0.801 (0.770) *N* = 124	− 1.207 (0.976) *N* = 143
Sig	*t*(3,441) = 3.75 *p* < 0.001	*t*(2,050) = 1.57 *p* = .115	*t*(6,943) = 5.17 *p* < 0.001	*t*(5,584) = 3.68 *p* < 0.001	*t*(5,624) = 4.56 *p* < 0.001
Cohen’s *d*	− 0.30	− 0.18	− 0.39	− 0.26	− 0.39
Non-UK graduates, female					
No ESCUW	− 0.615 (0.994) *N* = 3384	− 0.765 (0.866) *N* = 2056	− 0.490 (0.981) *N* = 4420	− 0.463 (0.916) *N* = 2876	− 0.466 (0.990) *N* = 2588
ESCUW	− 0.989 (1.009) *N* = 43	− 1.368 (0.865) *N* = 25	− 1.134 (0.814) *N* = 46	− 1.314 (0.715) *N* = 17	− 1.251 (0.861) *N* = 13
Sig	*t*(3,425) = 2.46 *p* = 0.014	*t*(2,079) = 3.46 *p* = 0.001	*t*(4,464) = 4.44 *p* < 0.001	*t*(2,891) = 3.82 *p* < 0.001	*t*(2,599) = 2.85 *p* = 0.004
Cohen’s *d*	− 0.38	− 0.70	− 0.66	− 0.97	− 0.79
Mean (range) Cohen’s *d*	− 0.52 (− 0.30; − 0.91)	− 0.53 (− 0.18; − 0.70)	− 0.49 (− 0.33; − 0.66)	− 0.48 (− 0.20; − 0.97)	− 0.41 (− 0.20; − 0.79)

### The causal relationship between examination performance and ESCUW sanctions

The previous analyses make clear that there is an association between poor examination performance and receiving ESCUW sanctions. The direction of causality though is not immediately clear as it may be that being investigated for FtP problems may itself have resulted in poor examination performance. We investigated the timing of exams and ESCUW sanctions for both the first attempt at MRCGP AKT and the first attempt at MRCP(UK) Part 1. The analyses were not carried out for MRCGP CSA and MRCP(UK) Part 2 and PACES as there were greater overlaps in the timing of ESCUW sanctions and the timing of the exam.

### MRCGP AKT

The first attempt at MRCGP AKT was a median of 4.81 years (mean = 6.68 years) after qualification, whereas the median date of a first ESCUW sanction was 9.59 years (mean = 11.10 years) after qualification, with the median interval between AKT and FtP sanction being 1.37 years (mean = 1.05 years). ESCUW therefore mostly postdates AKT. FtP procedures can though be slow, and we therefore considered AKT first attempt marks when the first attempt was at least 2 years *before* an ESCUW sanction (*n* = 162; mean standardised AKT score = − 0.751, SD = 1.049), those *within* 2 years of a sanction (*n* = 187; mean score = − 0.833, SD = 1.267) and those first attempts occurring two or more years *after* a sanction (*n* = 74; mean score = − 0.368, SD = 1.080). The differences between the groups were significant (one-way ANOVA, *F*(2,420) = 4.40, *p* = 0.013), but a post hoc test showed that the only difference was due to those taking AKT *after* an ESCUW sanction performing somewhat better.

### MRCP(UK) Part 1

The first MRCP(UK) Part 1 attempt occurred in a median of 1.88 years (mean = 1.88 years) after qualification, whereas the median date of a first ESCUW sanction was 10.95 years (mean = 11.87 years) after qualification, the median interval between Part 1 and FtP sanction being 4.56 years (mean = 4.21 years). In most cases, therefore, ESCUW clearly postdates Part 1 by a number of years. Because FtP procedures may be slow, we considered Part 1 first attempt marks at least 2 years *before* an ESCUW sanction (*n* = 292; mean standardised score = − 0.627, SD = 1.033), *within* 2 years of a sanction (*n* = 109; mean score = − 0.535, SD = 1.084) and two or more years *after* a sanction (*n* = 23; mean score = − 0.460, SD = 1.047). Differences between the groups in performance were not significant (one-way ANOVA, *F*(2,421) = 0.509, *p* = 0.602).

## Discussion

The present analyses show that UK doctors who subsequently have fitness to practise issues have previously performed less well at UK postgraduate medical examinations, with clinical assessments particularly seeming to relate to FtP sanctions. Our results are therefore compatible with previous studies such as that of Tiffin et al. [20] who showed that the GMC’s PLAB Part 1 (knowledge) and particularly Part 2 (skills) examinations predict subsequent sanctions in IMGs working in the UK. For the first time though, our study considers two of the major UK postgraduate examinations, which are taken by both UK graduates and IMGs, finding equivalent effects in both groups, and in particular, it demonstrates in all five assessments the linearity of the relationship between log_e_(OR) and examination performance over the entire range of examination performance.

Previous studies predicting fitness to practise, such as those using PLAB, have the advantage that licencing examinations are of necessity taken before clinical practice and censure can take place, making inference of the direction of causality straightforward. Postgraduate examinations though take place after doctors have been in clinical practice for a while, and therefore, causation might occur in the opposite direction, the stress associated with FtP investigations perhaps resulting in poorer examination performance. However, the analysis of timing in our study shows first attempts at MRCGP AKT and MRCP(UK) Part 1 are typically several years before an FtP sanction, and yet performance at AKT and Part 1 is still substantially poorer. The causal direction is therefore from poorer examination performance to FtP issues, rather than vice versa.

### Linearity, ‘good-enough’ and ‘more-is-better’

The large size of our study means that the detailed relationship between examination performance and log_e_(OR) for FtP sanctions can be examined (see Fig. 2). It is clear that the relationship is linear across the entire range of examination performance, as found elsewhere [17], every decrement in examination performance resulting in an equal increment in the log odds of being sanctioned. It should be mentioned that it is linearity on the odds scale which is relevant, and not linearity on a probability scale, probabilities having the problem that they cannot go below 0 or above 1 and therefore any relationship to ability must be non-linear, a difficulty that is not found on odds (logit) scales which are bounded by plus and minus infinity. It is for that reason that regression using binary variables uses a logistic (odds) scale.

**Fig. 3:**
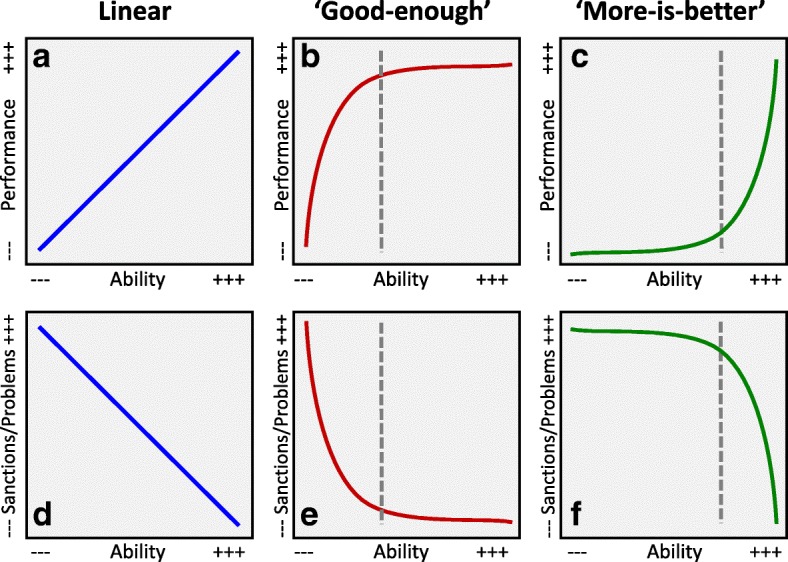
Expected relationships between performance (**a**–**c**) or sanctions/problems (**d**–**f***)* in relation to ability levels, for the linear (**a**, **d**), ‘good-enough’ (**b**, **e**) and ‘more-is-better’ models (**c**, **f**). See text for further details

Interpreting the linearity in Fig. 2 requires a consideration of what other form the relationship might have taken. An important approach to such issues is that developed by Arneson et al. [30] who presented what they call the “more-is-better” and the “enough-is-enough” models [30], although we use the terms slightly differently. The top row of Fig. 3, based on the approach developed by Arneson et al., shows how increasing ability might relate to increases in performance, whereas the bottom row shows the case discussed in this paper of how lower rates of sanctions or problems might relate to increasing ability. The linear model is the simplest and suggests that as ability increases, performance also increases in equal increments, with no apparent limit on that process. In contrast the ‘good-enough’ model in Fig. 3b, suggests, as Arneson et al. say, that ‘although higher scores are linked to better performance in the lower portion of the test-score range, there is some point at which the relationship levels off’ [30] (p.1336). The result is that the curve for performance is monotonically increasing but concave *downwards* (Fig. 3c), suggesting some sort of threshold, shown as a dashed grey line in the figure, beyond which there is little or no increase in performance with increasing ability. The model is a popular one, and Arneson et al. point out that it is used in Malcolm Gladwell’s best-selling book *Outliers* [31], where he cites Schwartz’s earlier model of selection in higher education, which had the title, ‘top colleges should select randomly from a pool of ‘good enough’’ [32], a proposal also put forward recently in a UK context [33]. The opposite of the ‘good-enough’ model is the ‘more-is-better’ model, which can be visualised as a monotonically increasing function which is concave *upwards*, as in Fig. 3c. At low performance levels, increasing ability has little effect on performance, but at high ability levels, increments in ability provide ever greater gains in performance. Once again, there is some sort of threshold, with performance below that level being almost uniformly weak. Finally, in the present case, where it is *low* rates of sanctions or problems which are desirable, the curves of Fig. 3a–c are inverted to give the curves of Fig. 3d–f.

Professional sanctions are particularly concerned with problems of patient safety. A common explanatory model is in terms of a few ‘bad apples’ who perform at very low levels and need to be weeded out (just as it is often erroneously claimed that most road traffic accidents are due to a minority of individuals with ‘accident-proneness’). That is, the ‘good-enough’ model in a different guise, the bad apples being below the threshold and responsible for most accidents. In contrast is a situation in which all doctors are vulnerable to problems, but increasing knowledge and skills gained by training mitigate against problems, as training shifts the distribution of knowledge and skills to the right and reduces the likelihood of problems at all levels of performance (see the figure 5 in a theoretical analysis by one of us [34]).

### Linearity emerges from encountering tasks with a wide range of difficulty levels

Which model applies linear, good-enough or more-is-better is primarily a statistical issue to be resolved with data, but extreme cases are easy to understand. Consider individuals who differ in mathematical ability across a wide range from innumerate to postgraduate. To work on a market stall selling fruit and vegetables, a knowledge of addition, subtraction, multiplication and (possibly) division is likely to be useful for charging customers and giving change. However, a knowledge of calculus, matrix algebra or Lie theory is unlikely to provide any additional benefit, so that the ‘good-enough’ model of Fig. 3b makes sense, with a threshold perhaps at about the typical level of a 10- or 11-year-old. In contrast, for post-doctoral study of elliptic functions, a very high level of mathematical attainment is essential, with even the ability of typical school leavers or graduates in mathematics hardly being able to help. In such a case, additional mathematical knowledge at higher levels is likely to be ever more useful in answering such arcane questions, so that ‘more-is-better’, with the minimal threshold probably being a doctorate in a relevant area. Finally, what about the wide range of mathematical applications that one encounters in everyday life, from simple to surprisingly complex? It seems reasonable to assume a linear model, in which with increasing mathematical ability, a wider range of quotidian problems can be solved. The linearity in a linear model emerges therefore from the summing of a series of good-enough or more-is-better models each with different thresholds, which correspond to different tasks in everyday life. Clinical practice can also be considered in such terms, a typical practitioner having a wide range of different tasks of different difficulties at which they need to remain competent, with serious failure in any likely to result in sanctions [35].

So, which model is appropriate for medical practice? Undoubtedly, there are advocates for ‘good-enough’ models, with arguments made that only knowledge of a core curriculum with only basic science relevant to frequently presenting conditions being necessary for doctors, the majority of anatomy or biochemistry or knowledge of a wide range of clinical conditions not being needed for safe, acceptable practice, at least under supervision. For high levels of specialisation and independent practice, that seems to be unrealistic, and specialist knowledge across a range of clinical and basic sciences seems ever more likely to be necessary. More is probably better, therefore, although more is perhaps not needed in all areas, making the linear model a good pragmatic compromise.

The form of the ability-performance relationship is relevant across many areas of medical education, as for instance in the current discussions on the UKMLA (UK Medical Licensing Assessment) which wishes to ‘[set] a common threshold for safe practice’ [36], the implicit assumption being that of the good-enough model. Often the setting of pass marks in examinations suggests that a threshold is being set on a good-enough model. However, it is highly doubtful that candidates scoring exactly at the pass mark are competent whereas those scoring one mark below the pass mark are not competent or that those exactly at the pass mark will perform as well in clinical practice as those in the top few percent of those passing the assessment (and just-passing candidates in postgraduate examinations clearly have problems with subsequent tests [22]). Pass marks are mainly for administrative and practical convenience, but are not really suggesting that ‘good-enough’ to pass is equivalent in all areas of subsequent practice. To paraphrase Cresswell [37], who draws on Sainsbury’s work on vagueness [38], a pass mark is merely a legal line which has only a pragmatic justification in an area where there are no actual boundaries.

The linearity of the relationships in Fig. 2 is now much more comprehensible. Not knowing enough results in poor performance, problems in practice and possible sanctions, whereas knowing more, and ever more, is a protection against problems and sanctions, particularly when practice involves a wide range of differing clinical presentations and appropriate actions. The linearity is therefore an important result as it emphasises that FtP issues are not merely problems associated with very poorly performing doctors (the ‘bad apples’). Even amongst high-performing doctors, additional increments in performance result in better performance and a reduction in the odds of FtP sanctions. A key implication is that any reduction of pass marks or standards in examinations, for whatever reason, perhaps political, pragmatic or otherwise, will result in higher subsequent rates of FtP issues as has been modelled elsewhere [39]. The corollary is that greater knowledge and higher performance are always of professional benefit, even in those who are already of high ability, as probably most high-performing professionals recognise.

### Clinical assessments vs knowledge tests

An important finding from the multiple regressions is that clinical assessments (CSA or PACES) predict FtP significantly better than do the knowledge assessments of AKT and Part 1. That result may seem surprising given the seemingly similar slopes of the graphs in Fig. 2. The difference arises because the simple regressions are just carried out for those candidates for whom data are available, whereas the multiple regressions are carried out for *all* candidates in the database, with imputation of missing values where needed. A pre-requisite of taking PACES (and Part 2) is having passed Part 1, and most candidates taking CSA will have passed AKT. There is therefore *restriction of range* in candidates taking later examinations, only better candidates taking them. However, what is required is a comparative assessment of construct validity on the basis of all candidates having taken all of the assessments, and for that, missing data need to be imputed. PACES predicts at a similar level as, say, Part 1, in the simple analyses, but the variance of the candidates is less for PACES, and if the likely ability level of all candidates is taken into account, then PACES will predict better across the entire range of ability. An alternative way of viewing it is that if just one test were to be applied to all candidates with the intention of predicting FtP issues, then PACES would predict better than Part 1. A separate approach to comparing knowledge-based and clinical assessments is our comparison of ROC curves, where the paired analysis is restricted to candidates taking both types of assessment and where the area under the curve is significantly greater for clinical as opposed to knowledge-based assessments, for both MRCGP and MRCP(UK).

### Conduct, trust and fitness to practise

Fitness to practise is at the core of being a doctor; doctors who are not fit to practise endanger patients and others. While doctors need to be competent, establish effective relationships with patients and act responsibly in relation to health care, those attributes, as the GMC makes clear, ‘while essential, are not enough’ [40]. The GMC continues,

‘Doctors have a respected position in society and their work gives them privileged access to patients … A doctor whose conduct has shown that [they] cannot justify the trust placed in [them] should not continue in unrestricted practice while that remains the case. In short, the public is entitled to expect that their doctor is fit to practise, and follows our principles of good practice described in *Good medical practice*’ [40] (paras 2-3).

Conduct and trust are therefore at the heart of FtP. That seemingly contrasts with the apparent content of postgraduate examinations such as MRCGP and MRCP(UK), for which knowledge, understanding and competency in practical skills are the essential components, most notably in ‘knowledge assessments’ such as MRCGP AKT and MRCP(UK) Parts 1 and 2 and ‘clinical assessments’ such as MRCGP CSA and MRCP(UK) PACES. Examination has neither conduct nor trust at its core, although most examiners would probably feel that those traits are implicit, but not explicit, in the assessments. It is therefore remarkable in many ways that poor performance in MRCGP and MRCP(UK) relates so strongly to FtP issues in our very large sample of doctors. A weakness of our study is that the LRMP does not specify the precise reasons for sanction (but these have been studied elsewhere [20], and future work should look in more detail at the grounds for sanction). Another potential weakness is that this study looked only at performance on an internal medicine or a general practice examination, whereas the likelihood of sanctions varies by specialty (including no specialty and dual specialty) [2]. That being said, many doctors take MRCP(UK) or MRCGP but do not complete their training in internal medicine or general practice, either moving into a different specialty or not completing their training, particularly since MRCP(UK) is an ‘entry’ exam. Furthermore, among doctors on the specialist and GP registers, physicians are among the least likely to be sanctioned whereas GPs are among the most likely. It is therefore likely that the large sample in our study covered a broad range of doctors working in a wide variety of specialties and clinical contexts.

Why performance in knowledge assessments and clinical assessments predict failures of the conduct and trust which are said to underpin FtP problems is not entirely clear and merits investigation. However, attaining appropriate levels of knowledge and competence does not come easily within professions such as medicine. Clinical practice itself helps, of course [41], but there is also an extensive need for doctors to read and study within their disciplines, to interact with colleagues as part of the social networks of knowledge that professions generate and to practise specific skills and competencies. All such things are driven as much by internal, intrinsic processes as by external forces and pressures, and the conscientiousness, the care and the concern which make doctors want to perform better and to know more are probably the same underlying psychological processes which generate trust and conduct that is appropriate. Medicine as a profession has never only been about pathophysiology and its vicissitudes, but instead is a continuum, for as Jonathan Miller put it, ‘one foot is planted in the physical world, electronic impulses and the muck of the human body; the other is planted in the subjective, experiential world of consciousness and conduct’ [42].

### Clinical examinations provide incremental validity for postgraduate examinations

The validity of postgraduate assessments is important, acting as a guarantor to the public and patients, as well as a professional justification for the time and effort expended by trainees on examinations. The clear relationship of poor performance to FtP issues in our five assessments, particularly taken in conjunction with other studies [16–18, 20], makes clear that postgraduate examinations are not mere academic games but are important evidence for the predictive validity of postgraduate examinations. A feature of UK postgraduate education is that examinations contain both knowledge assessments and clinical assessments, and our previous comparison of performance of candidates taking both MRCP(UK) and MRCGP suggests the knowledge and clinical assessments assess separate domains of expertise [21]. Our data suggest that clinical assessments are particularly important in predicting FtP, and clinical examinations almost always include communication skills (and as the GMC says, competence and communication are essential for doctors). That poor performance in the clinical examinations, CSA and PACES, is a better predictor of FtP problems than performance in knowledge assessments is of practical and political importance. A decline in clinical examination skills continues to be of concern [43, 44], despite many physicians finding examination important [45] and errors in patient care occurring because of poor examination skills [46–48]. Clinical examination in practice has been taking place less over the past four decades [49], perhaps as a result of an influential 1975 paper claiming that examination contributed little to diagnosis [50]. Postgraduate assessments in the UK (although rarely in the USA) emphasise clinical examinations as well as knowledge assessments. However, clinical assessments are expensive and time-consuming to run, needing experienced examiners in a one-to-one relationship with candidates who themselves are interacting with patients, be they surrogates or, in the case of MRCP(UK), real patients with real diseases and real symptoms and signs, meaning that examinations need to take place in a clinical environment. None of that comes cheap, and there is always a temptation to assume that computer-based assessments might replace traditional clinical examinations, but it may well be mistaken, particularly if poor performance at clinical assessments particularly relates to FtP problems. That has implications for postgraduate examinations, undergraduate examinations and licencing examinations such as USMLE Stage 2 CS and the still being developed UKMLA.

## Conclusions

This study is the first to demonstrate for UK postgraduate examinations that knowledge assessments and clinical assessments are independent predictors of FtP sanctions, with clinical assessments showing a larger predictive effect than knowledge assessments (as was also previously found for PLAB assessments of IMGs [20]). The greater predictive validity of clinical assessments over knowledge assessments has implications for the design of undergraduate and postgraduate clinical examinations. Likewise, the linearity of the effects across the entire range of knowledge and skills assessments implies that additional knowledge and skills are beneficial to doctors at all levels of attainment.

## Abbreviations

ABIM: American Board of Internal Medicine
AKT: Applied Knowledge Test
ANOVA: Analysis of variance
AUC: Area under the curve
BME: Black and Minority Ethnic
CSA: Clinical Skills Assessment
DIF: Differential item functioning
EM: Expectation maximisation
ESCUW: Erasure, Suspension, Conditions on Practice, Undertakings or Warnings
FtP: Fitness to practise
GMC: General Medical Council
GP: General practice or general practitioner
IBM SPSS: IBM Statistical Package for the Social Sciences
IMG: International medical graduate
LRMP: List of Registered Medical Practitioners
MAR: Missing at random
MCQ: Multiple-choice question
MPTS: Medical Practitioners Tribunal Service
MRCGP: Membership of the Royal College of General Practitioners
MRCP(UK): Membership of the Royal Colleges of Physicians of the United Kingdom
NBME: National Board of Medical Examiners
NHS: National Health Service
NIHR: National Institute for Health Research
nPACES: New Practical Assessment of Clinical Examination Skills
OR: Odds ratio
PACES: Practical Assessment of Clinical Examination Skills
PLAB: Professional and Linguistic Assessments Board
PMQ: Primary medical qualification
R: The R project for statistical computing
RCGP: Royal College of General Practitioners
ROC: Receiver operating characteristic
SD: Standard deviation
UCL: University College London
UK: United Kingdom
UKMLA: United Kingdom Medical Licensing Assessment
USMLE: United States Medical Licensing Examination

## References

[1] General Medical Council (2017). Doctors who commit suicide while under GMC fitness to practise investigation.

[2] Unwin E, Woolf K, Wadlow C, Dacre J (2014). Disciplined doctors: does the sex of a doctor matter? A cross-sectional study examining the association between a doctor's sex and receiving sanctions against their medical registration. BMJ Open.

[3] General Medical Council (2017). Securing the licence to practise: introducing a Medical Licensing Assessment a public consultation.

[4] Archer J, Lynn N, Coombes L, Gale T, de Bere SR (2016). The medical licensing examination debate. Regul Gov.

[5] Flier LA, Henderson CR, Treasure CL (2016). Time to eliminate the Step 2 Clinical Skills examination for US medical graduates. JAMA Intern Med.

[6] General Medical Council (2017). An introduction to revalidation.

[7] General Medical Council (2017). The GMC’s fitness to practise procedures.

[8] Ludka-Stempien K, Woolf K, McManus IC (2013). Poor performance on the MRCP(UK) examination predicts license limitations in subsequent medical practice.

[9] Ludka-Stempien K (2015). The predictive validity of the MRCP(UK) examination (PhD thesis).

[10] Royal College of Anaesthetists (2017). A report on the welfare, morale and experiences of anaesthetists in training: the need to listen.

[11] Messick S (1995). Validity of psychological assessment: validation of inferences from persons’ responses and performances as scientific inquiry into score meaning. AP.

[12] Downing SM (2003). Validity: on the meaningful interpretation of assessment data. Med Educ.

[13] Lissitz RW (2009). The concept of validity: revisions, new directions, and applications.

[14] Kane MT (2014). Validating the interpretations and uses of test scores. JEM.

[15] American Educational Research Association, American Psychological Association, National Council on Measurement in Education (2014). Standards for educational and psychological testing.

[16] Norcini JJ, Boulet JR, Opalek A, Dauphinee WD (2014). The relationship between licensing examination performance and the outcomes of care by international medical school graduates. Acad Med.

[17] Papadakis MA, Arnold J, Blanton H, Holmboe ES, Lipner RS (2008). Performance during internal medicine residency training and subsequent disciplinary action by state licensing boards. Ann Intern Med.

[18] Cuddy MM, Young A, Gelman A, Swanson DB, Johnson DA, Dillon GF (2017). Exploring the relationships between USMLE performance and disciplinary action in practice: a validity study of score inferences from a licensure examination. Acad Med.

[19] Cuddy MM, Winward ML, Johnston MM, Lipner RS, Clauser BE (2016). Evaluating validity evidence for USMLE step 2 clinical skills data gathering and data interpretation scores: does performance predict history-taking and physical examination ratings for first-year internal medicine residents?. Acad Med.

[20] Tiffin PA, Paton LW, Mwandigha LM, McLachlan JC, Illing J (2017). Predicting fitness to practise events in international medical graduates who registered as UK doctors via the Professional and Linguistic Assessments Board (PLAB) system: a national cohort study. BMC Med.

[21] Wakeford R, Denney ML, Ludka-Stempien K, Dacre J, McManus IC (2015). Cross-comparison of MRCGP & MRCP(UK) in a database linkage study of 2,284 candidates taking both examinations: assessment of validity and differential performance by ethnicity. BMC Med Educ.

[22] McManus IC, Ludka K (2012). Resitting a high-stakes postgraduate medical examination on multiple occasions: nonlinear multilevel modelling of performance in the MRCP(UK) examinations. BMC Med.

[23] McManus IC, Chis L, Fox R, Waller D, Tang P: Implementing statistical equating for MRCP(UK) Parts 1 and 2**.** BMC Med Educ 2014, 14**:** 204 http://www.biomedcentral.com/1472-6920/14/204; doi:10.1186/1472-6920-14-204. 10.1186/1472-6920-14-204 PMC4182791 25257070

[24] Royal College of General Practitioners (2017). MRCGP examinations.

[25] Elder A, McAlpine L, Bateman N, Dacre J, Kopelman P, McManus IC (2011). Changing PACES: developments to the examination in 2009. Clin Med.

[26] General Medical Council (2015). The state of medical education and practice in the UK: 2015.

[27] Hope D, Adamson K, McManus IC, Chis L, Elder A (2018). Using differential item functioning to evaluate potential bias in a high stakes postgraduate knowledge based assessment. BMC Med Educ.

[28] R Core Team (2017). R: a language and environment for statistical computing.

[29] Robin X, Turck N, Hainard A, Tiberti N, Lisacek F, Sanchez F-C (2011). pROC: an open-source package for R and S+ to analyze and compare ROC curves. BMC Bioinformatics.

[30] Arneson JJ, Sackett PR, Beatty AS (2011). Ability-performance relationships in education and employment settings: critical tests of the more-is-better and good-enough hypotheses. Psychol Sci.

[31] Outliers: the story of success. New York: Little, Brown; 2008.

[32] Schwartz B (2005). Top colleges should select randomly from a pool of ‘good enough’. Chron High Educ.

[33] Major LE, Machin S (2018). Social mobility and its enemies.

[34] McManus IC, Vincent CA, Lens P, van der Waal G (1997). Can future poor performance be identified during selection?. Problem doctors: a conspiracy of silence.

[35] Handfield-Jones RS, Mann KV, Challis ME, Hobma SO, Klass DJ, McManus IC (2002). Linking assessment to learning: a new route to quality assurance in medical practice. Med Educ.

[36] General Medical Council (2018). Medical Licensing Assessment.

[37] Cresswell M (2003). Heaps, prototypes and ethics: the consequence of using judgments of student performance to set examination standards in a time of change.

[38] Sainsbury Mark (2003). Departing from Frege.

[39] Davison I, McManus IC, Taylor C (2016). Evaluation of GP Specialty Selection [Report commissioned by Health Education England].

[40] General Medical Council. The meaning of fitness to practise. Essays in the philosophy of language. London: General Medical Council; 2014. https://www.gmc-uk.org/-/media/documents/dc4591-the-meaning-of-fitness-to-practise-25416562.pdf.

[41] McManus IC, Woolf K, Dacre J, Paice E, Dewberry C (2013). The academic backbone: longitudinal continuities in educational achievement from secondary school and medical school to MRCP(UK) and the Specialist Register in UK medical students and doctors. BMC Med.

[42] Bassett K. Operating theatre. CAM. 1995:(14)7–11.

[43] Jauhar S (2006). The demise of the physical exam. NEJM.

[44] Elder A, Verghese A (2015). Bedside matters – putting the patient at the centre of teaching and learning. J R Coll Physicians Edinb.

[45] Elder A, McManus IC, Patrick A, Nair K, Vaughan L, Dacre J (2017). The value of the physical examination in clinical practice: an international survey. Clin Med.

[46] Reilly BM (2003). Physical examination in the care of medical inpatients: an observational study. Lancet.

[47] Verghese A, Charlton B, Kassirer JP, Ramsey M, Ioannidis JPA (2015). Inadequacies of physical examination as a cause of medical errors and adverse events: a collection of vignettes. AJM.

[48] Verghese A, Horwitz RI: In praise of the physical examination [Editorial]. Brit Med J 2009, 339b5448. 10.1136/bmj.b5448 20015910

[49] Oliver Charlotte M, Hunter Selena A, Ikeda Takayoshi, Galletly Duncan C (2013). Junior doctor skill in the art of physical examination: a retrospective study of the medical admission note over four decades. BMJ Open.

[50] Hampton JR, Harrison MJG, Mitchell JRA, Prichard JS, Seymour C (1975). Relative contributions of history-taking, physical examination, and laboratory investigation to diagnosis and management of medical outpatients. Brit Med J.

